# Motion Sensors for Knee Angle Recognition in Muscle Rehabilitation Solutions

**DOI:** 10.3390/s22197605

**Published:** 2022-10-07

**Authors:** Tiago Franco, Leonardo Sestrem, Pedro Rangel Henriques, Paulo Alves, Maria João Varanda Pereira, Diego Brandão, Paulo Leitão, Alfredo Silva

**Affiliations:** 1 Research Centre in Digitalization and Intelligent Robotics (CeDRI), Polytechnic Institute of Bragança, 5300-253 Bragança, Portugal; 2 ALGORITMI Centre, University of Minho, 4800-058 Braga, Portugal; 3 Federal Center of Techonology of Rio de Janeiro (CEFET/RJ), Rio de Janeiro 20271-204, Brazil; 4 INOVA+, 4450-309 Porto, Portugal

**Keywords:** IMU sensor, algorithmic complexity, knee angle, muscle rehabilitation, wearable system

## Abstract

The progressive loss of functional capacity due to aging is a serious problem that can compromise human locomotion capacity, requiring the help of an assistant and reducing independence. The NanoStim project aims to develop a system capable of performing treatment with electrostimulation at the patient’s home, reducing the number of consultations. The knee angle is one of the essential attributes in this context, helping understand the patient’s movement during the treatment session. This article presents a wearable system that recognizes the knee angle through IMU sensors. The hardware chosen for the wearables are low cost, including an ESP32 microcontroller and an MPU-6050 sensor. However, this hardware impairs signal accuracy in the multitasking environment expected in rehabilitation treatment. Three optimization filters with algorithmic complexity O(1) were tested to improve the signal’s noise. The complementary filter obtained the best result, presenting an average error of 0.6 degrees and an improvement of 77% in MSE. Furthermore, an interface in the mobile app was developed to respond immediately to the recognized movement. The systems were tested with volunteers in a real environment and could successfully measure the movement performed. In the future, it is planned to use the recognized angle with the electromyography sensor.

## 1. Introduction

  Over human life, our body goes through several muscular and hormonal changes. Generally, a healthy person reaches the peak of their strength and muscle mass between 25 and 34 years old. Afterwards, the human body slows down the metabolism, hormonal cycles, and muscle recovery. The intensification of these symptoms is usually reported at 50 years old and can lead to disorders such as sarcopenia or degenerative diseases such as Knee Osteoarthritis (KOA) [1].

The World Health Organization (WHO) reports [2] that the recovery and maintenance of functional capacity are one of the main concerns for healthy aging, especially since the worsening of symptoms caused by pathologies related to aging, such as KOA, can compromise human locomotion capacity, requiring the help of an assistant and reducing independence [3]. In addition, the world’s elderly population is growing fast, with 1 billion elderly people living now, which is 2.5 times greater than in 1980.

In the search for new treatments that can better fit the needs of elderly patients who suffer from muscular disabilities, the NanoStim project emerges. The NanoStim project aims to reduce the burden on healthcare services by developing a solution that allows electrostimulation treatment to be performed at the patient’s home. Nowadays, the treatment with electrostimulation for muscle strengthening is divided into sessions performed in a physiotherapy clinic, requiring two or three visits per week for a session that lasts 40 min to complete.

For this treatment at home to be feasible, the architecture of an electronic computer-controlled system was designed, including a wearable component capable of applying an electrostimulation protocol defined by the physician [4]. The wearable technology was chosen due to its unique advantages of instantaneity, flexibility, and the ability to transport sensors easily [5]. Thus, in addition to allowing the treatment to be performed at home, it is also possible to track biophysical and biomechanical signals during a treatment session and use the data acquired to adjust the stimulation protocol considering the particularities of each patient.

In order to understand which sensors can bring relevant information to the proposed treatment, a literature review [6] was conducted looking for studies that used biomechanical data to classify the stages of KOA and raise characteristics that contribute to the pathology interpretation. As a result, two sensors were highlighted as the most significant in identifying distinctions between the motion behavior of patients, the Electromyographic (EMG) sensor and the Inertial Measurement Unit (IMU) sensor.

EMG sensors can monitor the electrical activity of a muscle during a given movement or activity through electrodes placed on the surface of the skin. This information can be essential for muscle rehabilitation treatment, providing metrics to adjust stimulation parameters relative to muscle effort during a treatment session. In addition, the use of electromyography can be considered common in physiotherapy clinics, reducing the learning curve of professionals in interpreting the proposed treatment. As part of the NanoStim project, the implementation of a wearable system capable of acquiring EMG signals and performing electrostimulation simultaneously can be found in the article [7].

The knee angle is the most significant parameter in the KOA classification, mainly due to the difference in the behavior of the lower limbs in everyday activities, such as walking. The studies conducted reported that the behavior of patients tends to present a similar pattern depending on the stage of the pathology. In addition to the use of cameras, the most used method to acquire the knee angle was through two IMU sensors. The first sensor is positioned on the patient’s thigh, and the second is on the shin; thus, the angle of each sensor is correlated to calculate the knee angle.

Likewise, the knee angle also becomes relevant in our context, expanding our ability to classify the progress of a treatment based on the difference in behavior recorded over the sessions. In addition, with the streaming data, it is possible to verify if the movement performed by the patient during the treatment is correct and to act if it is not.

myHealth is a mobile app for Android designed to offer a technological interface between the patient and the physician in an electrostimulation treatment at home. This application under development was able to apply a stimulation protocol and collect data from the EMG sensor simultaneously using the aforementioned wearable system. More details about the myHealth app and the communication with the wearable system during a treatment session can be seen in article [8].

In order to understand the movement performed during a treatment session, recognizing the knee angle, we propose in this article two approaches that are implemented in the myHealth app. The first approach implements two wearable modules to perform the acquisition of the IMU sensor and transmit the data via Bluetooth Low Energy (BLE). The second approach implements knee angle recognition with streaming data in a mobile application.

This present article proposes the following contributions:

a The development of a low-cost wearable system capable of acquiring data from an IMU sensor;
b Identification of the information needed to calculate the knee angle and the construction of an interface to recognize knee movement in a mobile app;
c Characterization and comparison of low-cost computational filters to improve the accuracy of motion sensors.

This document contains six more sections. Section 2 discusses related works, presenting similar applications found in the literature; Section 3 describes the proposed solution, including the system architecture, the electrical circuit, and the communication protocol. Section 4 explores the mathematical model used to calculate the angles from the IMU sensors. Section 5 presents the algorithms and tests performed to correct sensor reading errors. Section 6 describes the steps followed to implement knee angle recognition in the mobile application called myHealth. Section 7 reports the main conclusions and future work.

## 2. Literature Review

  Currently, it is possible to find comprehensive literature using motion sensors during rehabilitation sessions. In a literature review [9] on the topic focused on technological and clinical advances, evidence is reported that indicates a potential benefit for pathologies such as stroke, movement disorders, knee osteoarthritis, and running injuries.

Similar to the objective of the NanoStim project, Sultan [10] carried out a study applying an electrostimulation treatment in patients with KOA through a wearable device and a mobile application. The wearable used was capable of collecting Range Of Motion (ROM) values through two accelerometers. However, the acquired data were not used in the treatment, only stored by the app. After the end of the treatment session, the data were sent to the cloud and made available for the physician to monitor the progress. Although the patient was required to adjust stimulation intensity and treatment duration, the treatment showed an improvement in ROM values and a significant reduction in pain scores.

Gait analysis is another way of measuring body movements, body mechanics, and the activity of the muscles. In [11], Milic employed an evaluation of the gait parameters to understand metabolic and mechanical variables. For this study, a tool called Optogait was used. This tool can display all of the collected data in real-time through a software platform and is paired with lateral and sagittal video analysis. In this study, the Optogait system was positioned on a treadmill where the candidate walked, and it was possible to observe the parameters of the gait cycle in real-time. The system also provides feedback regarding movement asymmetries and what can be employed for clinical intervention. The authors demonstrated that the proposed Iso-Efficiency Speeds (IES) method offers the highest performance benefits while lowering or at least not increasing the metabolic cost. Although this study employs a deterministic analysis due to equations for uphill walking gait, the results are concise and in accordance with the literature’s desired outputs.

Machine Learning (ML) techniques are also being explored to improve the diagnosis using data from IMU sensors. Mezghani [12] creates a dataset from a commercial device capable of recording the knee angle in search of mechanical biomarkers. In this study, it was possible to classify the KOA stages using the Kellgren and Lawrence scale with 85% accuracy. Similarly, Kobsar [13] also uses IMU sensors to classify KOA using ML. This study proposes the creation of a wearable device and tests different positions for gait identification. Although the study did not specifically use the knee angle, it was able to reach 81.7% in the classification.

Despite this, in the systematic review [14] on the accuracy of clinical applications using wearable motion sensors, it is reported that it is difficult to estimate the reliability of the studies. This is because many of the studies explored use different and sometimes inadequate methods, making the task of correlating the real advances achieved by this technology inaccurate. In addition, the studies found generally do not offer a detailed explanation of how the wearable system works, especially the integration with sensors.

In this line, the study reported by Almeida [15] contributed to our work reported in this article. The authors developed a wearable acquisition system capable of collecting data from the IMU sensor and transporting the data collected via Wi-Fi to a client system developed in Python. The study develops a proof of concept by comparing three correction filters (Complementary, Kalman, and Madgwick) in three different scenarios. The authors made the code and libraries used for the tests performed available on GitHub to make it possible to replicate the wearable system. The study points out Madgwick as the filter with a lower error percentage, followed by the Kalman and Complementary filters.

Despite this, the authors processed the data in the cloud and did not consider the computational cost of the filtering algorithms and the amount of data required for transmission. This can be a problem in embedded systems, as processing power is generally very low, and transmission technologies such as Bluetooth Low Energy allow for the exchange of small data packets.

## 3. Wearable Acquisition System

  The IMU is a type of wearable technology that can be employed to measure motion biomechanics [16]. The IMU sensor is usually composed of an accelerometer and a gyroscope, both with three axes (*x*,*y*,*z*); it is also possible to find models that include a magnetometer. The metrics collected from accelerometer sensors, such as the magnitude of an acceleration, loading rate, and shock attenuation, are similar to metrics obtained using force plates [17]. When the gyroscope and/or magnetometer sensors in an IMU are used, the acquired results provide information on the kinematics, including segment and joint rotations [18].

One IMU model commonly employed in wearable applications is the MPU-6050. This device offers low power consumption, low cost, and high-performance requirements for smartphones, tablets, and wearable sensors [19]. With its ability to precisely and accurately track user motions, the MPU-6050 allows MotionTracking technology to convert handsets and tablets into powerful 3D intelligent devices that can be employed in health monitoring applications [20]. Furthermore, this IMU device has a three-axis gyroscope, three-axis accelerometer, a Digital Motion Processor^TM^ (DMP), and a dedicated I^2^C sensor bus, all in a small package $\left(4\times 4\times 0.9\right)\;mm$ [19].

Regarding the sensor precision, the MPU-6050 features three 16-bit Analog-to-Digital Converters (ADCs) for digitizing the gyroscope outputs and three 16-bit ADCs for digitizing the accelerometer outputs, which allows it to track both fast and slow motions. In addition, the parts feature a user programmable gyroscope full-scale range of ±250, ±500, ±1000, and ±2000${}^{\circ }/s$ and a user-programmable accelerometer full-scale range of ±2g, ±4g, ±8g, and ±16g [20].

**Figure 1:**
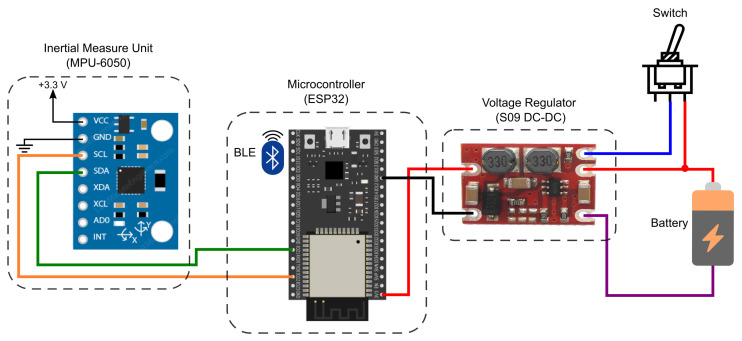
IMU acquisition system diagram.

To take advantage of the functionalities available in the MPU-6050, an architecture based on the ESP32 Microcontroller Unit (MCU) was designed, as illustrated in Figure 1. The proposed module comprises a battery, a DC–DC voltage regulator, MCU, and the IMU sensor. The employed source was a conventional 3.7 V 700 mA h lithium-ion battery. The voltage regulator used was the S09 model due to its low cost and output voltage level, which allows an input voltage of (3–15) V, output voltages of 3.3 V/4.2 V/5.0 V/9.0 V/12.0 V, and maximum output current of 0.6 A. The MCU was selected as the ESP32 due to its low cost and the availability of wireless communication protocols (Wi-Fi and BLE), which provide the I^2^C bus to collect the acquired data from the MPU-6050 and sends the collected data through BLE to a mobile application.

**Figure 2:**
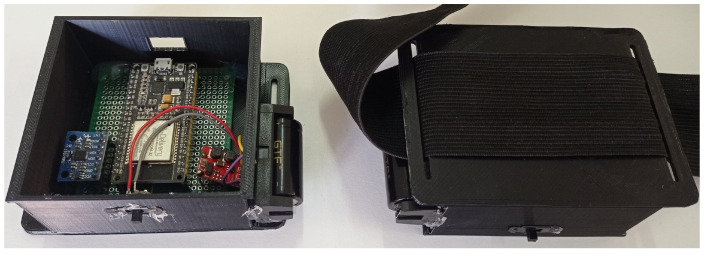
The electronic components soldered inside the case.

From the defined scheme, the electronic components were soldered on a universal perforated board and fixed with hot glue in a case developed in a 3D printer. The case illustrated in Figure 2 was designed to protect the electronic components and provide a way to tie the wearable system to a surface, such as a person’s thigh. Thus, to keep the case stable in the desired location, it has made two gaps on each side of the case, making it possible to pass clothing elastics and make a knot.

ESP32 is an MCU with enough computational power to acquire sensor data at high frequencies since the clock of a standard model, such as *ESP32-WROOM-32D*, is above 150 MHz. However, the wearable system under development will not only collect data from the IMU sensor, but the software will also be responsible for performing the following tasks simultaneously: (1) collect data from the EMG sensor, (2) receive the stimulation protocol, (3) apply the stimulation, and (4) transport all collected data via BLE to the mobile app.

Considering the scenario described, the wearable systems, capable of performing the electrostimulation sessions, Ref. [7] were refactored with the addition of IMU data acquisition. To simulate the designed treatment activities, the refactored software was installed in one of the wearable modules. For the second module, new software was implemented and programmed to only acquire the data from the IMU and transmit it via BLE. With this, it is possible to carry out the tests considering the real scenario expected by the software and the hardware, manifesting resource limitations of the computational power.

**Figure 3:**
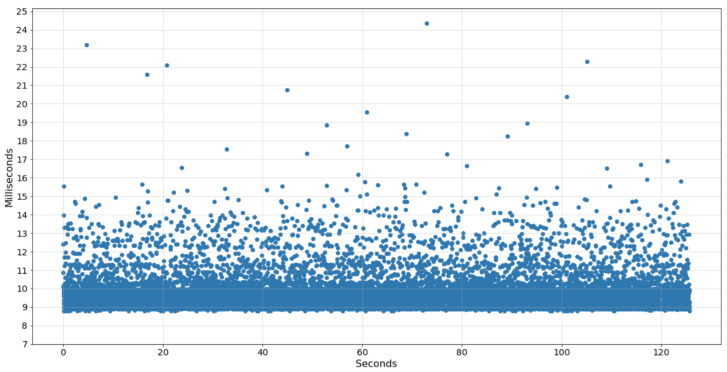
Δ Time of IMU samples collected by the wearable system.

Given this, the data acquisition of the IMU sensor was implemented in a thread programmed to collect a sample every 8 ms, ideally resulting in a sampling frequency of 125 Hz. However, due to the sharing of processing power with the other tasks, the data collected showed acquisitions of 99–100 Hz. Furthermore, the average time interval between each collection was 9.8 ms, with some peaks above 24 ms. The chart in Figure 3 displays the time difference between each sample collected by the wearable system in an example of an acquisition performed.

BLE was chosen for the data transmission resource because it presents a lower power consumption than standard Bluetooth and Wi-Fi, significantly increasing battery life. However, in addition to being expensive in terms of processing power, version 4.2 of the BLE limits the amount of data per packet to 517 bytes. As a result, to send all the raw data acquired by the IMU sensor, it is necessary to transmit at least 200 data packets per second to keep the mobile app synchronized. This is because the raw data includes six variables of 16 bytes for the accelerometer and gyroscope and one more variable of 4 bytes for the time interval, resulting in 100 bytes per sample.

Optimally for the proposed solution, it would only be necessary to send the sensor angle in relation to the sagittal plane of the human body. Each processed angle sample requires 4 bytes: 1 byte for the sign, 2 bytes for the integer value, and 1 byte for the mantissa. Thus, it is possible to send up to 125 samples per packet in more than one second of data acquisition.

The implemented strategy consisted of sending an IMU data packet every 400 ms, essentially aiming to make the mobile app more synchronized. Each data packet has a vector with three variables of 4 bytes each: the calculation of the roll angle, the value of the X-axis of the gyroscope, and the time interval. Thus, every 400 ms, an average of 40 data samples are generated, resulting in 480 bytes. In this way, it is possible to transmit all the data collected up to that moment, and the maximum would be 43 samples (516 bytes) per packet.

To start and finish data collection, the mobile application that can receive the EMG signal during a treatment session [8] was also refactored to receive the IMU data. Thus, during the session, the application processes and stores the biofeedback data in an internal file. When the session ends, the app sends the file to the cloud via an HTTP API, according to the system architecture [4].

## 4. Mathematical Model

**Figure 4:**
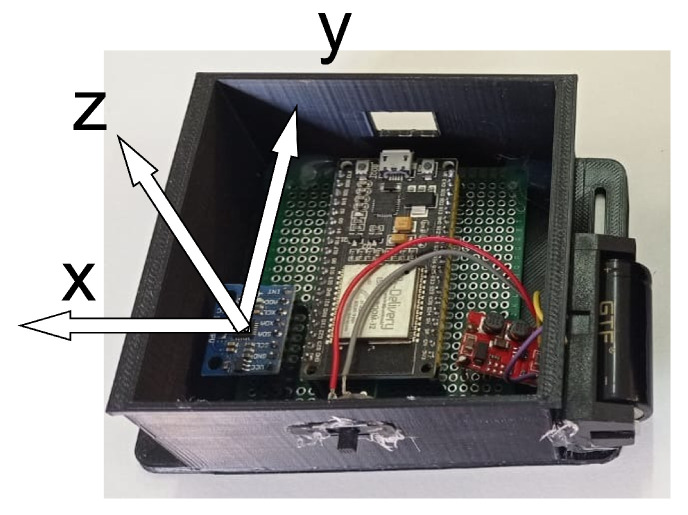
Sensors with the reference coordinate system.

  The Mathematical model used in this work is based on the theory of multibody system dynamics, as presented by Olinski et al. [21]. In this approach, the orientation of a body in space is given by the orientation of a local frame attached to the body with respect to a reference coordinate system (Figure 4).

**Figure 5:**
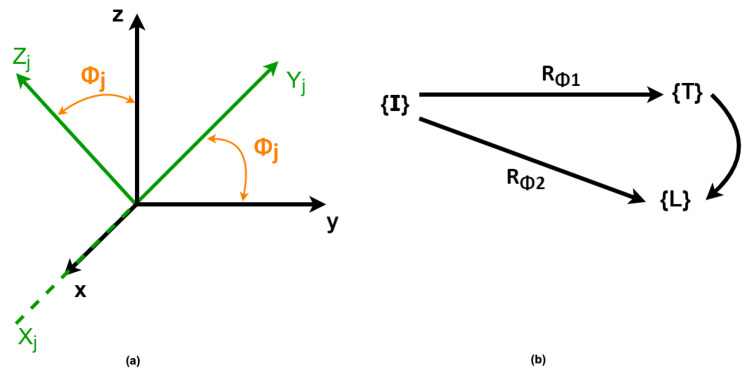
(**a**) Frame representation with respect to a reference frame. (**b**) Linear mapping of a frame to another.

Considering the particular case in which the orientation changes occur in a specific plane, as presented in Figure 5a, the mapping of frames with respect to a reference frame can be represented by a single rotation from a reference to another (Figure 5b).

To map a frame worth in respect to another in the case of rotations in the YZ-plane of an angle $\Phi_{j}$ around the x-axis, as presented in Figure 5, the Euler angles are determined by applying the linear mapping successively from rotation matrices ($R_{\Phi 1}$ and $R_{\Phi 2}$) in each of these spaces. The rotation matrix is given by Equation (1), where j=1 or j=2:

(1) $$R_{j}=\left[\begin{array}{ccc} 1 & 0 & 0\\ 0 & cos(\Phi_{j}) & -sen(\Phi_{j})\\ 0 & sen(\Phi_{j}) & cos(\Phi_{j}). \end{array}\right]$$

**Figure 6:**
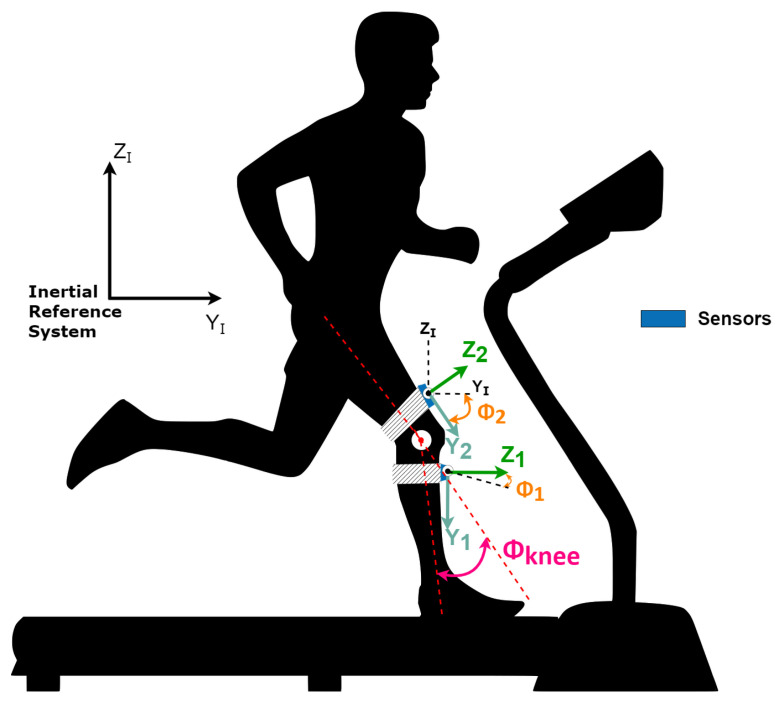
Sensors with the angles representation. Adapted from [21].

Considering the sagittal plane as the plane of reference for the movements, once the abduction/adduction angles are neglected (Figure 6), the orientation of the leg’s frame and the thigh’s frame, both concerning the inertial coordinate system, are represented as rotations in the referred plane.

Once the monitored movement happens in the sagittal plane, the knee angle is given by the difference between the thigh and leg angles $\Phi_{knee}=\Phi_{2}-\Phi_{1}$.

Once the IMU’s data are measured with respect to the inertial coordinate system, the attachment of an IMU to each body permits the determination of their orientation naturally with respect to the inertial system, and the difference between them gives the knee angle.

## 5. Acquisition Optimization

  As described in Section 3, IMU sensors such as the MPU-6050 model can capture a given movement with reasonable accuracy and sensitivity. However, the performance presented in the real environment shows inconsistency and electrical noise in the acquired samples. Thus, to improve the accuracy of the collected data, two preliminary activities will be described: the calibration of the sensors to remove the offset values and the optimization using a low-cost computational filter that will be implemented in the wearable system.

### 5.1. Calibration

Although the IMU sensors are already calibrated by their manufacturers, over time, it is possible to record measurements that are completely different from zero when the sensor is static, as reported by Woodman et al. [22]. To align the measurements to zero, the following steps were performed:

1. Position the wearable modules on a straight surface;
2. Turn on the modules and wait for 1 min;
3. Send calibration command from the mobile app;
4. The Wearable modules start acquiring IMU data at the maximum executable frequency for 10 s;
5. The average of the acquired values is calculated for each axis of the accelerometer and gyrocospe;
6. The resulting values are saved in the internal memory of the wearable system.

When the wearable system is powered on again, the stored values, known as offset values in our system, will be accessed. Thus, for each sample acquired, the offset value of the respective axis will be subtracted, resulting in an approximate measurement of zero when the module is static. In step two, the modules remain on for one minute before calibration to stabilize the modules and to ensure that the temperature sensor included in the MPU-6050 model does not interfere in the readings.

### 5.2. Filters with Algorithmic Complexity O(1)

Within the Signal Processing area, several filters have been studied to improve the accuracy of IMU sensors. As commented in the literature review, implementing filters such as Kalman and Madgwick can considerably reduce errors in measuring joint angles. However, as described by Valade [23], implementing filters such as Kalman’s in embedded systems requires a very high computational cost.

The Big O notation is one of the most used notations to describe the computational cost of a given algorithm. This notation takes into account the size of the input and counts the number of instructions used to execute a given sequence of code. For example, for an algorithm that calculates whether the given input is even or odd, only one instruction will be used, resulting in an algorithmic complexity of O(1). For an algorithm that needs to traverse a vector of size n, the algorithmic complexity is O(n), since at least n instructions will be executed to complete the task [24].

The algorithm complexity presented by Valvede [23] for the Kalman filter is O(10*n*^3^); for the extended Kalman filter, it is O(4*n*^3^). A study on the algorithmic complexity of the Madgwick filter was not found, but as calculations with matrices were used, the algorithmic complexity was to be at least O(n). Furthermore, these algorithms require memory resources to store the intermediate matrices needed in every calculation.

Therefore, three filters with the lowest computational cost with algorithmic complexity of O(1) will be tested: Simple Moving Average (SMA), Exponentially Moving Average (EMA), and Complementary Filter of the accelerometer and gyroscope (CF). The algorithm that presents the best results will be implemented in the wearable’s embedded system to run at the time of collection and transmit only the values of the angles to the mobile app.

Moving average filters have the ability to smoothen out the oscillations presented in a signal. In this article, the signal is preset by a sequence of numbers ordered by time, also known as a time-series array. To calculate SMA, it is first necessary to define the only required parameter, the window size (*w*) to be moved along the vector. Thus, for each sample acquired, the simple average is calculated among all the last *w* elements of the array with the new sample included.

It is possible to apply the SMA filter by traversing a vector between the defined window to find the new average, resulting in an algorithmic complexity of O(w). However, to optimize the calculation, a variable was allocated to store the sum of the momentary window. Thus, for each sample it will be necessary to update the sum value and divide by the parameter *w* to calculate the new SMA. Algorithm 1 displays the instructions executed to calculate the SMA.   
**Algorithm 1:** Instructions for updating the SMA.
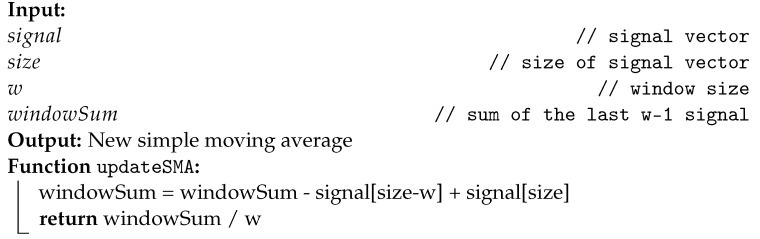


The calculation of the conventional average assumes that all elements have the same weight. The weighted average is the calculation of the average by assigning non-equivalent weights to the elements of the defined window. The EMA filter smoothens the signal by calculating the weighted average considering exponential factor (α). The factor α is expressed between 0 and 1 and represents how much the oldest samples should contribute to the result. The EMA filter algorithm can be implemented recursively to optimize processing [25], requiring only one instruction to perform the calculation, described in Algorithm 2.   
**Algorithm 2:** Instructions for updating the EMA.
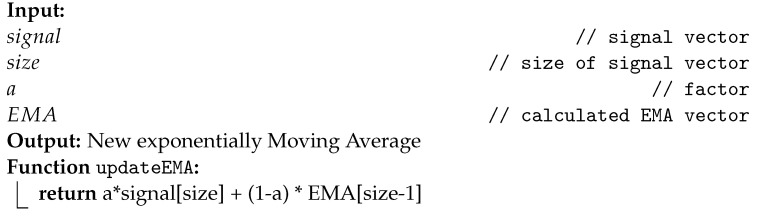


Similar to EMA, the only adjustable parameter to calculate the CF is factor α. However, in this case, the factor α refers to the share of participation between the accelerometer and the gyroscope to compose the angle measured, hence the complementary in the filter name. Complementary filters can be applied whenever there are two or more sources of the same information. For example, Almeida [15] used the angle calculated through the magnetometer sensor to compose the CF as a third source.

**Figure 7:**
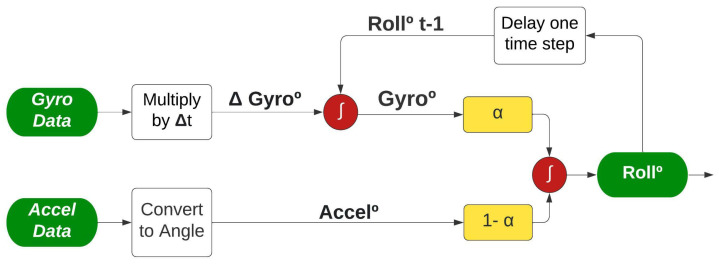
Complementary Filter applied to IMU sensor, adapted from [27].

From the signal processing point of view, the data acquired by the accelerometer can better measure the slower and static movements, so a low-pass filter is applied. The data acquired by the gyroscope can better measure faster and more dynamic movements, so a high-pass filter is applied [26]. The filters are combined through the factor α, resulting in an integrated signal as illustrated in Figure 7.

As shown in Figure 7, the two angles (Accel° and Gyro°) are calculated differently before being combined to improve the angle measurement (Roll°). For the accelerometer route, no additional information is required, it is only necessary to convert the accelerometer acquired data to an angle; the equation is described in Section 4. On the other hand, the gyroscope data require an extra step; the value acquired is multiplied by the time interval between the current and past sample, resulting in the angular shifting described as Δ Gyro. Afterwards, Δ Gyro is added to the angle found in the last sample collected, representing the past angle being corrected considering the last movement measured.

The CF algorithm can also be optimized and implemented recursively, as demonstrated by Albaghdadi el al. [27]. This algorithm requires only two instruction lines to run, as described in Algorithm 3. Unlike the other two algorithms presented, CF requires the storage of one more variable, the Δ time between samples, represented by (dt).   
**Algorithm 3:** Instructions for updating the CF.
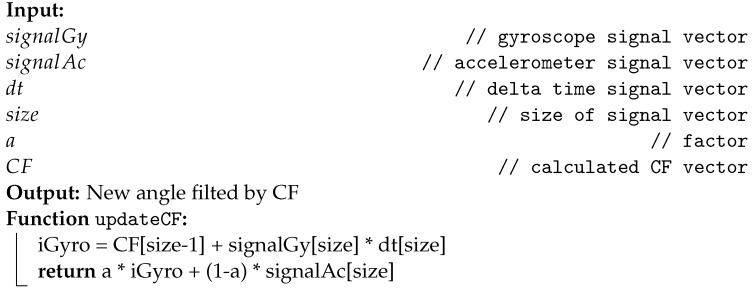


### 5.3. Test Scenario

To investigate which optimization filter could give the best performance, the UR3 robotic arm was used. The UR3 collaborative industrial robot from Universal Robotics is suitable for assembly and screwdriver activities, usually positioned on the top of benches [28]. This robotic arm was chosen for two main reasons: firstly, the availability of the robot in the laboratory where the research was carried out; secondly, due to the fact that the UR3 has a certificate validating the precision of the joint’s movements. Thus, it is possible to configure the UR3 to perform a given movement with a minimum error, making the validation more cohesive.

**Figure 8:**
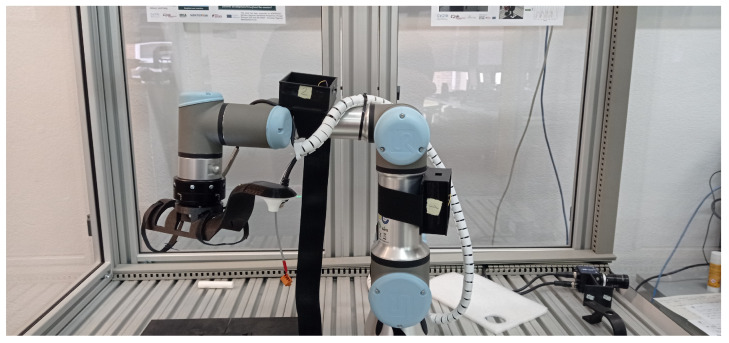
Wearable modules attached to the UR3 robot.

Each wearable module was attached with clothing elastics to different parts in one of the joints of the UR3 robot, representing the knee joint, as illustrated in Figure 8.

The UR3 robot can be programmed to follow a sequence of positions determined by the joint angle. Thus, for all tests performed, the robot was programmed to move the selected joint in the following positions repeatedly: $\left[0^{\circ },90^{\circ },75^{\circ },90^{\circ },60^{\circ },45^{\circ }\right]$. Each time the joint reached one of the chosen angles, the UR3 remained static for two seconds before moving to the next position.

**Figure 9:**
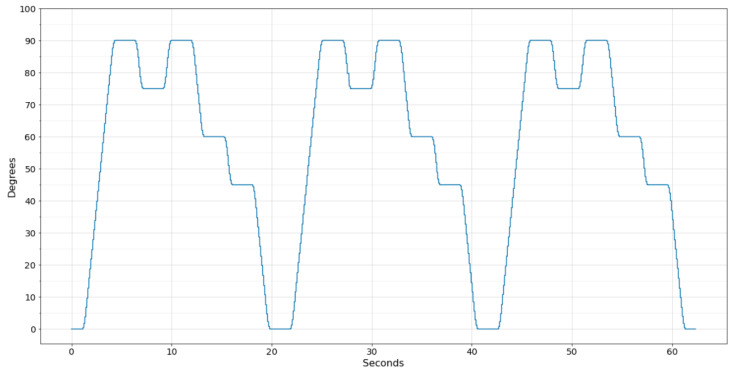
Movement performed by the UR3 robot.

To compare the movement performed by the UR3 with the wearable system, a python application was developed to communicate with the UR3 via Wi-Fi and collect the angle of the chosen joint. However, the UR3 updates the register that stores the joint angle at a frequency of 30 Hz, and the sampling frequency of the developed software was set to 100 Hz to approximate the amount of data generated by the wearable system. Figure 9 displays the data collected from the UR3 robot performing the programmed movement three times.

To analyze the data acquired by the wearable system, a treatment session without stimulation was recorded for each test performed. In this way, the acquired data became available in a MongoDB (MongoDB available on the website: https://www.mongodb.com accessed on 10 September 2022) database, where it can be imported into a Jupyter Notebook (Jupyter Notebook available on the website: https://jupyter.org accessed on 10 September 2022) for analysis before implementing the feature of knee angle recognition in the mobile app. Finally, two time-series arrays were generated for each test, containing the angles registered by the UR3 and the wearable system.

Three main metrics were used to evaluate the performance of the optimization filters: Mean Absolute Error (MAE), Mean Squared Error (MSE), and Root Mean Squared Error (RMSE). All the metrics seek to parameterize the difference between the two vectors acquired.

To better describe the evaluation metrics, the UR3 vector will be interpreted as the vector of actual points *A*. After applying one of the optimization filters, the wearable system vector will be interpreted as *F*. If all elements of vector *A* are subtracted from vector *F*, the result is an error vector *E*, in which the sum is expected to be 0 if there is no error.

The MAE metric is the simple calculation of the average of errors, however, disregarding the error signals. Thus, the wearable system angles that were above or below the correct angle have the same weight in the calculation. Equation (2) presents the calculation of the MAE metric:

(2) $$MAE=\frac{1}{n}\sum_{t=1}^{n}\left|A_{t}-F_{t}\right|$$

The second evaluation metric is the RMS, which calculates the average of squared errors. Thus, the errors are potentiated in comparison to the MAE metric, making the differences between the errors more significant. Equation (3) describes the calculation of the RMS metric:

(3) $$MSE=\frac{1}{n}\sum_{t=1}^{n}{\left(A_{t}-F_{t}\right)}^{2}$$

Lastly, the RMSE is the calculation of the standard deviation for the error vector *E*. This metric highlights the largest errors (outliers), significantly increasing the value of the metric. It is possible to calculate the RMSE from the square root of the MSE metric, as described in Equation (4):

(4) $$RMSE=\sqrt{\frac{1}{n}\sum_{t=1}^{n}{\left(A_{t}-F_{t}\right)}^{2}}=\sqrt{MSE}$$

### 5.4. Experimental Results

From the defined scenario, the systems were turned on, and data were acquired for 100 s. The same test was performed several times to verify the consistency of the acquired data, and no significant differences were found. The results presented in this session are a clipping of the movement performed by the systems three times during one of the acquisitions.

**Figure 10:**
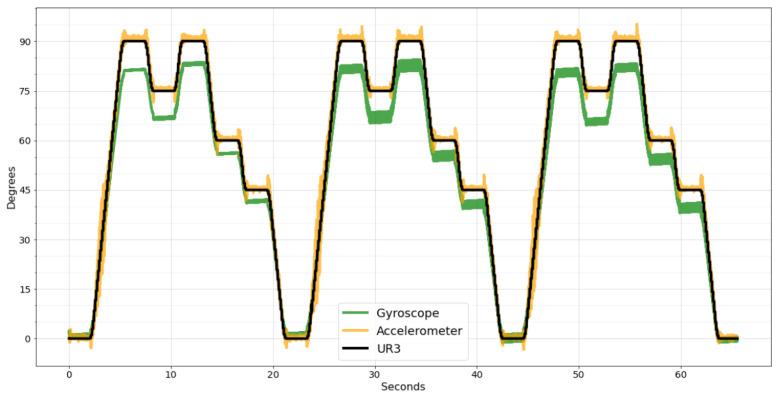
Wearable system raw data synchronized with UR3 data.

Although the two systems were set up with the same sampling frequency, it was impossible to enable data acquisition from both systems simultaneously, making it necessary to synchronize the data manually. Figure 10 shows the raw data of the wearable system synchronized with the data acquired by the UR3 robot.

As can be seen in Figure 10, both sensors, gyroscope, and accelerometer can reproduce the movement performed by the UR3, but they are inaccurate. As the accelerometer can better measure slow movements, the error is small during continuous movements, as shown in Figure 10 in the range of motion from 0° to 90°. However, when the UR3 stops, the accelerometer takes time to stabilize, generating noise peaks.

Contrarily, the gyroscope can better measure sudden speed changes, presenting less noise. However, in continuous movements, a small error is accumulated in every sample, resulting in a significant difference compared to the desired degree at the end of the movement.

Comparing the two sensors using the evaluation metrics, the gyroscope has a considerably worse result than the accelerometer. For the MAE metric, the accelerometer has a value of 1.27 and the gyroscope 5.37, four times higher. For the RMSE, the accelerometer has 2.09 and the gyroscope 6.18, three times higher. Lastly, the MSE of the accelerometer is 4.38, and for the gyroscope it is 38.27, eight times higher. Due to this significant difference in precision, the SMA and EMA filters were applied only to the accelerometer data since the data cannot be combined in these two filters, and the result with gyroscope data would be worse.

The three chosen filters have only one parameter to be adjusted to extract the maximum efficiency from each filter. In order to find the optimal point of these parameters, the range of interest that each parameter can vary was first chosen. Being [1, 15], steps of 1, for the window *w* of the SMA; [0, 0.5], steps of 0.01, for the factor α of the EMA; [0, 1], steps of 0.01, for the factor α of the CF.

**Figure 11:**
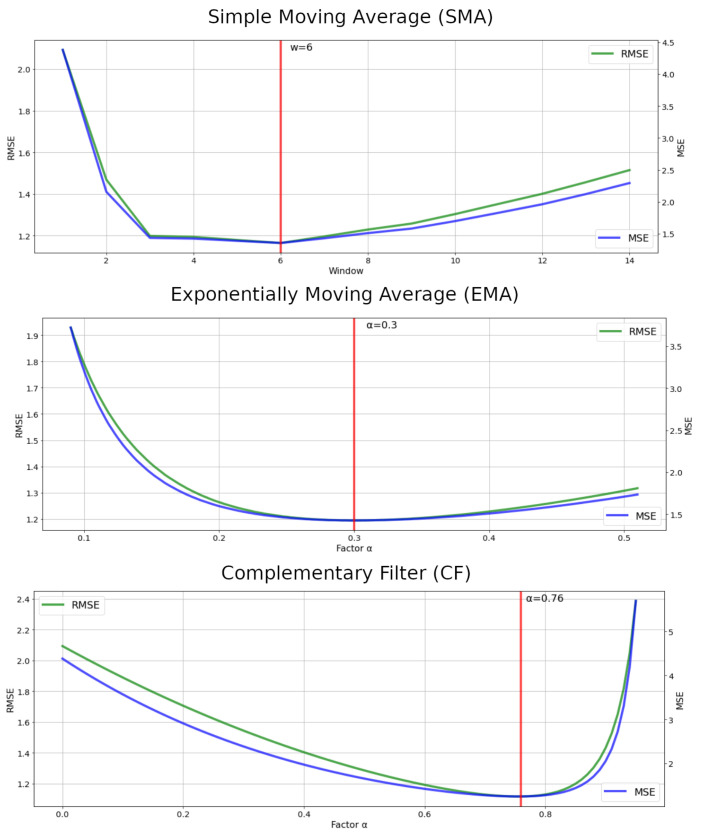
Optimal points of the adjustable filters parameters.

After, the filters were applied to the wearable system’s raw data, varying between the possibilities of the defined ranges. Each time a filter was applied, the evaluation metrics were calculated, resulting in the comparative chart in Figure 11.

The red vertical lines in Figure 11 show the optimal points for each parameter defined by the lowest RMSE and MSE values. With this, it is possible to find out which of the selected filters had the smallest error in measuring the movement of the UR3 robot. Table 1 displays the calculated values of the evaluation metrics for the wearable system’s raw data and the three filters applied SMA, EMA, and CF.

As seen in Table 1, the three filters applied improved the wearable system’s raw data error in more than 42% of the RMSE and 67% of the MSE. The MAE metrics of all filters were below 1 degree. The CF filter obtained the best result among the filters, but with a slight difference of 4% over the EMA filter and only 2% over the SMA in RMSE and MSE.

Despite this, the same test was performed more than once, and the CF filter always obtained better results than the other filters. Because of this, the CF filter was selected to be implemented in the wearable embedded system. Thus, the mobile app will only receive the angles already processed, reducing the amount of information needed to be transmitted and optimizing the BLE packages.

**Figure 12:**
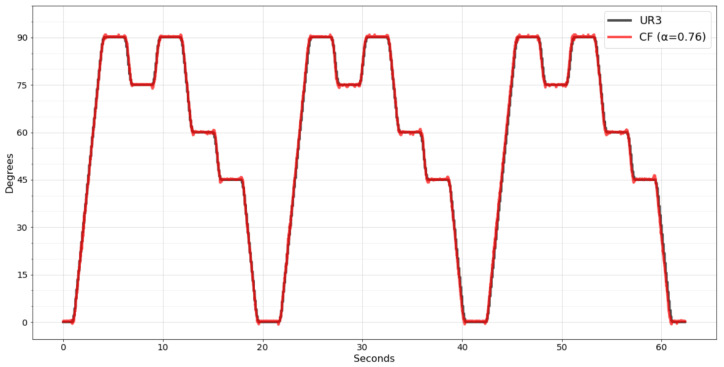
CF filter implemented in wearable embedded system.

Figure 12 shows the movement recognized by the wearable system with the CF filter already implemented. Analyzing the new data, it was noticed that the performance of the CF filter was even better, resulting in 0.6 degrees on MAE, 1.0058 on MSE, meaning a 77% improvement, and 1.0029 on RMSE, meaning a 52% improvement.

It was possible due to the improved resolution of the worked data. As previously, the raw data were sent to the mobile app; the maximum that the mantissa could reach was 255 since only one byte was reserved. Once the calculation is performed within the embedded system, it is possible to declare variables with greater precision to perform the calculation, such as the double type, representing a mantissa of up to 15 decimal places.

## 6. Mobile App Implementation

The myHealth app was developed by Franco et al. [8] for patients to manage their electrostimulation treatment sessions at home. For this, the app has a communication protocol with the wearable system capable of simultaneously sending the stimulation rules and collecting the EMG sensor data during a session. The protocol is flexible to adjust the stimulation parameters during the session, enabling the app to consider the patient’s immediate response through the sensors.

The mobile app has three main screens for interacting with the patient, the first being the home screen on which the history of treatment sessions and upcoming sessions are displayed. When the patient selects a session to perform, the monitoring treatment screen will be displayed. In this interface, it is possible to follow the treatment progress, command the treatment with pause and stop and see a real-time chart of the EMG signal. After the session ends, a feedback form screen displays some questions about the patient’s physical status.

All information generated during a treatment session is made available to the physician responsible for the care plan. With this, the physician will be better prepared to design the next treatment session, paying attention to the last session’s performance and the treatment’s progress.

Knee angle recognition will be one of the essential attributes within this context. In addition to being sent to the physician, it will also be helpful to guide the patient to follow the treatment instructions and confirm that the movement is consistent with the desired one. As already described in Section 3, the app was refactored to receive the IMU sensor data and transport them to the cloud along with the EMG sensor data. With the optimization filter implemented, the wearable system sends only the angles in a packet with approximately 40 samples every 400 ms, resulting in a sampling frequency of 100 Hz.

In order to have an immediate graphical response to the recognized movement, the session monitoring screen has been refactored by adding a new tab called IMU.

**Figure 13:**
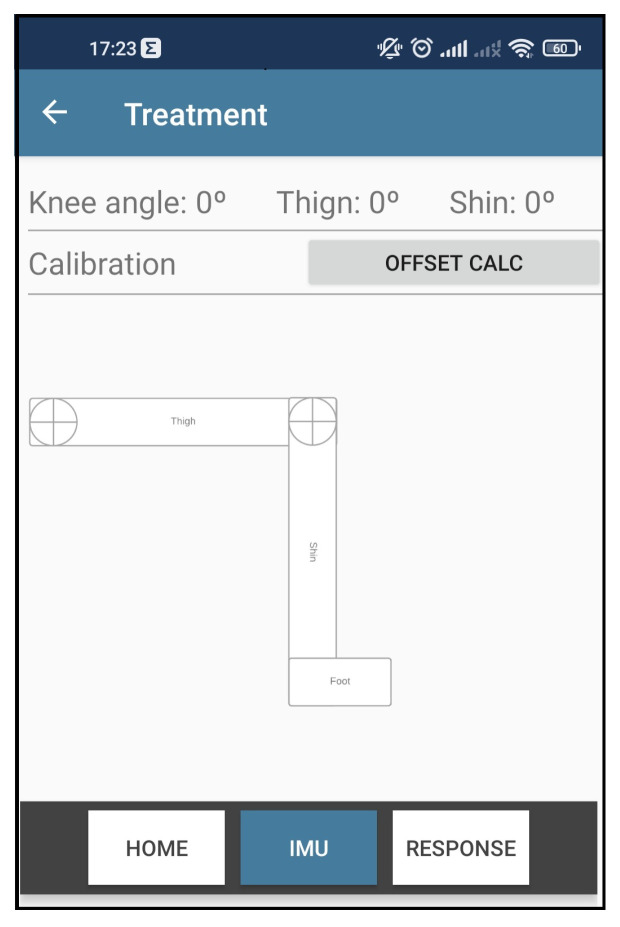
Knee angle recognition app interface.

As can be seen in Figure 13, in this new interface, it is possible to visualize the thigh angle, the shin, and the resulting angle of the knee. A button was created to send the command calibration to the wearable system to facilitate the tests. Lastly, a set of rectangles was designed to represent the patient’s thigh, shin, and foot members. Thus, when the mobile app receives the data packet from the wearable system, the interface updates the rotation of the rectangles representing the movement identified.

With the implementations, the wearable modules were put back in the UR3 robot, and the UR3 motion sequence was executed again. This test verified that the rectangles representing the patient’s leg could follow the movement performed by the UR3. The performance remained the same since there were no more changes in the algorithms, only in the graphical part of the app.

### Test on Volunteers

The wearable module systems were applied to a real environment to validate the developed content. Five volunteers were recruited to perform a test performing the knee extension movement. In this test, each volunteer remained seated on a bench and was requested to lift their leg five times for one minute, keeping their leg raised for approximately 4 s each time in the maximum muscle contraction.

**Figure 14:**
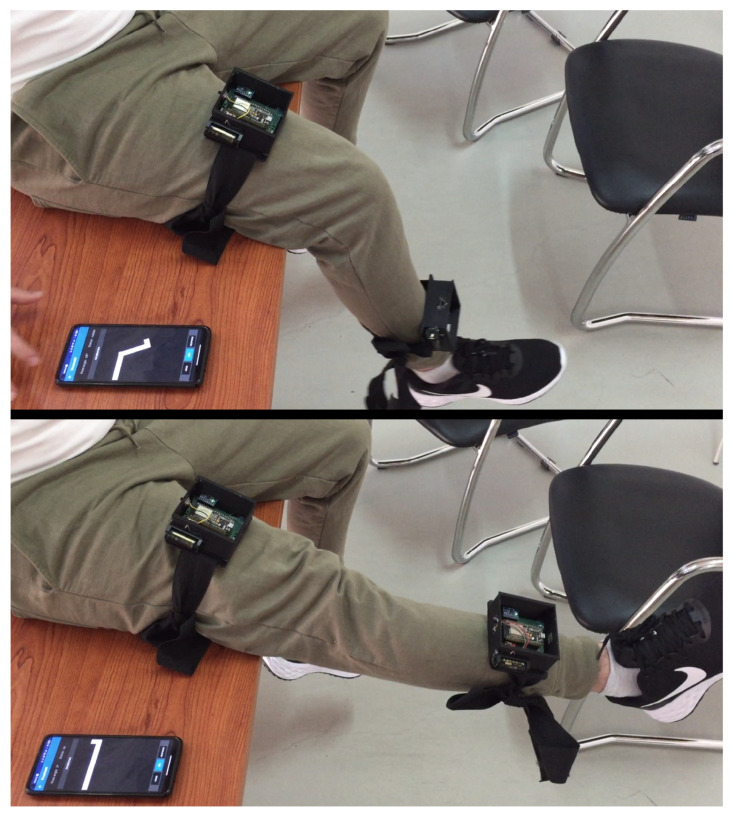
Wearable system and mobile app recognizing the movement of a volunteer’s leg.

Figure 14 shows the volunteer’s knee angle being recognized by the MyHealth mobile app through the IMU sensors of the wearable system developed.

As planned, the app successfully recognized the volunteer’s knee angle. However, it was noted that one more adjustment would need to be added to the systems. As can be seen in Figure 14, the wearable module positioned on the volunteer’s thigh does not remain precisely parallel to the thigh bone. Thus, it generates an angular deviation that varies between each person due to the unique anatomy of the leg muscle.

Fortunately, this angular deviation tends to be constant and the same during all treatment sessions, varying only by person. Thus, this value can be added and validated by the physician during the first treatment sessions, which are expected to be performed in the clinic and accompanied by the professional.

**Figure 15:**
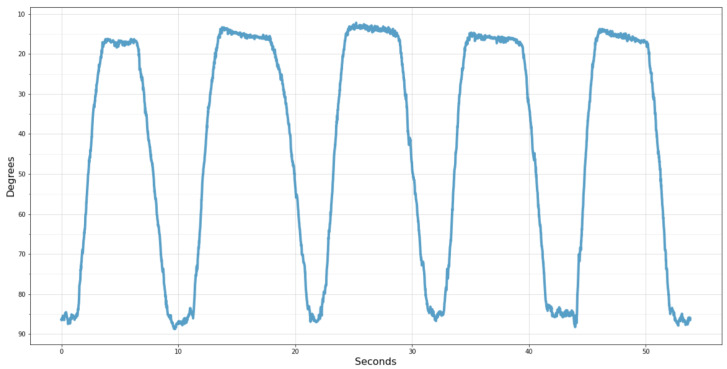
Knee angle acquired by the wearable system.

Finally, an example of the information that will be delivered in the administrative portal to the physician after each treatment session can be seen in Figure 15. In this case, the chart shows the volunteer’s knee angle movement during the test performed.

For better understanding, the scale was inverted, with 90° representing the thigh and shin aligned perpendicularly and 0° when the leg is fully extended. Thus, it becomes more evident after the five times that the volunteer raised his leg, remained elevated, and lowered it repeatedly for approximately 10 s.

## 7. Conclusions

This article presents the design, the necessary hardware, and the implementation of a wearable motion acquisition system. This acquisition system is composed of two main components, an ESP32 microcontroller and an MPU-6050 sensor (IMU) and its development is low cost. Due to this, this hardware can present limitations in some environments, as in the case reproduced in this article. The system implemented in the wearable’s hardware was designed to share its processing time to acquire data from an EMG sensor, apply stimulation, and send data to the mobile app. With this in mind, the wearable system was developed with the primary requirement to consume the minimum processing power to collect the data from the IMU sensor.

The focus of the system implemented in the wearable is to provide the data necessary for a mobile app to recognize the leg’s movement through the knee angle. According to this objective, three filters were explored to reduce the noise of the IMU sensor data, namely, SMA, EMA, and CF. The CF filter produced the best values among the evaluation metrics, achieving an improvement of 77% in the MSE and 52% in the RMSE in relation to the raw data. The wearable acquisition system showed an absolute average error of 0.6 degrees in recognizing the movement performed by the UR3 robot arm.

The recognition of the knee angle will be an asset for the myHealth app, as its objective is to enable performing a treatment with electrostimulation at the patient’s home. For this purpose, an interface was developed to show a sketch of the patient’s leg with its components. As soon as myHealth receives new data from the wearable system, the leg representation is recalculated according to the recognized movement, and a fresh sketch is redrawn, exhibiting the patient’s response to the stimuli.

We plan to refactor this interface in the future to show instructions interactively during a treatment session. This way, the treatment may be more attractive to elderly audiences. In addition, all movement performed by the patient during treatment is saved and made available for the physician to analyze later.

The system was tested with volunteers in a real environment and successfully measured the movement performed. Due to the heterogeneous anatomy of the volunteers, it was realized that incorporating an initial parameter would be needed. This parameter refers to angular deviation in a static position. This information should be measured and validated by the physician during the first treatment sessions, which are expected to occur in the physical therapy clinic.

In addition, the presented system has some limitations and restrictions to operating as planned. The system can measure the angle of only one knee joint at a time, so to measure both knees, it would be necessary to duplicate the IMU sensors and refactor the communication between the wearable system and the mobile app. Although the two modules can measure the angle regardless of the surface placed, the calculations performed in the mobile app assume that the modules are in the correct order and positioned on the same axis of the body, with the sagittal or frontal axis being possible. In this way, if the modules have been switched or twisted, the mobile app will misinterpret the measured movement.

In the near future, it is expected to embed the wearable modules presented in this article on a Printed Circuit Board (PCB). The PCB designed for the NanoStim project includes an EMG sensor and an electrostimulation actuator. With this in mind, the IMU sensors will be incorporated into a wearable, such as pants or shorts, and, through two cables connected to the PCB, responsible for power supply and data communication. Thus, it is expected to solve the positioning constraint, as the sensors will be positioned and fixed on the wearable in the correct and unalterable order.

With the complete hardware, it will be possible to test all the components designed to carry out a treatment session simultaneously and synchronized. As an example of a more complex validation, it can be the use of the Ariel Performance Analysis System (APAS), which uses cameras to measure the movement of the joints, providing a more reliable benchmark for the presented system.

Furthermore, we plan to create algorithms that can benefit from the combination of IMU and EMG sensors. An example is implementing a contraction clipping algorithm, using the knee angle to indicate the initial and final moment that the EMG sensor data should be clipped. With the EMG sensor alone, identifying contraction can be tricky since injured or diseased muscles can present low activity in the EMG signal. 

## Figures and Tables

**Table 1:** Evaluation metrics of the applied filters in degrees.

	ACC	GYRO	CF (α = 0.76)	SMA (*w* = 6)	EMA (α = 0.3)
**Min**	0.0001	0.0078	0.0003	0.0002	0.0016
**Max**	15.553	12.6619	6.477	7.7324	7.649
**MAE**	1.2795	5.3728	0.8775	0.907	0.9225
**RMSE**	2.0922	6.1867	1.1173	1.1645	1.1942
**MSE**	4.3806	38.2753	1.2484	1.356	1.4262
**Improved ACC RMSE**			46.59%	44.34%	42.92%
**Improved ACC MSE**			71.5%	69.04%	67.44%

## Data Availability

Not applicable.
